# Do Individuals Aged 50 or Older View Cognitive Conditions Differently Than Physical Conditions? Evidence From a Pooled Analysis of Illness Perceptions in Type 2 Diabetes and Mild Cognitive Impairment

**DOI:** 10.1093/geroni/igad027

**Published:** 2023-04-13

**Authors:** Hyejin Kim, Jennifer H Lingler, Catherine M Bender, Steven M Albert, Susan M Sereika

**Affiliations:** Department of Adult Health and Gerontological Nursing, Rush University College of Nursing, Chicago, Illinois, USA; Department of Health and Community Systems, University of Pittsburgh School of Nursing, Pittsburgh, Pennsylvania, USA; Alzheimer’s Disease Research Center, University of Pittsburgh, Pittsburgh, Pennsylvania, USA; Department of Health and Community Systems, University of Pittsburgh School of Nursing, Pittsburgh, Pennsylvania, USA; Department of Behavioral and Community Health Sciences, School of Public Health, University of Pittsburgh, Pittsburgh, Pennsylvania, USA; Department of Health and Community Systems, University of Pittsburgh School of Nursing, Pittsburgh, Pennsylvania, USA

**Keywords:** Age, Alzheimer’s disease, Chronic disease, Diabetes complications, Perception

## Abstract

**Background and Objectives:**

Type 2 diabetes (T2DM) and mild cognitive impairment (MCI) are common late-life physical and cognitive health conditions. Illness perceptions, an individual’s personal beliefs about the conditions, should be explored in the context of disease characteristics (physical or cognitive). This secondary analysis explored illness perceptions with a priori hypotheses about control (perceived controllability) and coherence (perceived understanding) dimensions among persons with T2DM and MCI, treating each as an exemplar of late-life physical and cognitive health conditions. We also explored whether age, education, and comorbid conditions moderate the relationships between T2DM or MCI condition groups and illness perceptions.

**Research Design and Methods:**

This cross-sectional, descriptive study examined baseline data collected from 146 T2DM to 90 MCI participants in 2 independent studies. The 9-item Brief Illness Perception Questionnaire was used to identify the similarities and differences in illness perceptions among persons with T2DM and MCI. We performed hierarchical linear regression controlling for identified covariates.

**Results:**

We found that T2DM and MCI participants had significantly different illness perceptions, including perceptions of personal control (*b* = −0.943, *p* = .009), treatment control (*b* = −1.619, *p* < .001), and coherence (*b* = −1.265, *p* = .001), after controlling for covariates. The results suggest that persons with MCI were likely to believe that their condition is less controllable (through their own strategies or medical treatment) and less understandable compared with their T2DM counterparts. Such associations remained statistically significant when the interactions were added to the models.

**Discussion and Implications:**

As T2DM and MCI are prevalent late-life conditions, health care professionals should consider individuals’ subjective perceptions about their conditions in the context of disease characteristics when counseling secondary prevention strategies for disease management. Further research on illness perceptions in other conditions is needed to ensure the replicability of our findings.

**Translational Significance:** Despite the exponential increase of age-related chronic conditions, knowledge of individuals’ beliefs about their conditions (i.e., illness perceptions) in the context of disease characteristics (physical or cognitive) remains limited. The findings of similarities and differences in illness perceptions among persons with type 2 diabetes and those with mild cognitive impairment, as examples of late-life physical and cognitive conditions, demonstrate the significance of understanding illness perceptions in the context of disease characteristics. Given that illness perceptions affect self-management behaviors, this study will accelerate the translation of research on disease management in age-related chronic conditions into clinical practice.

## Background and Objectives

The increasing number of older adults living with chronic conditions ( Boersma et al., 2020) has major public health implications. Older adults are afflicted with various types of age-related chronic conditions, falling into two broad categories, those which affect physical health (e.g., type 2 diabetes mellitus [T2DM], hypertension, and arthritis) or those which affect cognitive health (e.g., mild cognitive impairment [MCI], Alzheimer’s disease, and other related dementias [ADRD]). There is an overlap between the two types of conditions, such as the importance of self-management and their association with mental health (e.g., depression and anxiety; Ma, 2020; Park & Reynolds, 2015). Yet, persons with chronic conditions may hold distinct views regarding what they perceive as physical versus cognitive health conditions. For example, the relatively greater stigma associated with cognitive conditions (e.g., MCI and ADRD) as compared with physical conditions (e.g., T2DM and hypertension) may drive differences in perceptions about the conditions among those affected. Ultimately, such perceptions may affect self-management behaviors (e.g., physical activity, healthy diet) for health promotion.

Indeed, T2DM and AD are among the most prevalent age-related physical and cognitive conditions in the United States (Centers for Disease Control and Prevention, 2022; National Council on Aging, 2021). Although T2DM and early-stage cognitive impairment (i.e., MCI) may be asymptomatic or unnoticeable to the persons affected, lifelong management to prevent T2DM complications and AD development is necessary for both conditions. Although both T2DM and MCI may be asymptomatic or unnoticeable to the persons affected, lifelong management to prevent complications is necessary for both conditions. Despite the common features of the conditions, a diagnosis of MCI is relatively ambiguous in the context of its etiology, treatment, or prognosis compared with T2DM, which may make persons with MCI feel uncertain about their condition. In particular, although T2DM can be well managed by maintaining a healthy lifestyle or taking medications, MCI, as a potential precursor to AD, has no definitive therapies to cure or manage the disease, suggesting that thoughts and beliefs about the condition and its symptoms among persons with MCI may be unlike those with T2DM.

Research shows that individuals’ thoughts and beliefs about their diagnosis or disease-related symptoms, referred to as *illness perceptions*, affect disease self-management behaviors (Hagger et al., 2017; Leventhal et al., 2003, 2016). The *Common Sense Model (CSM) of Self-Regulation* (Leventhal et al., 1984, 2003) is an empirically validated framework that explicates a set of individuals’ illness perceptions comprising eight dimensions: (1) *identity* (i.e., perceived disease-related symptoms), (2) *consequences* (i.e., perceived impact of the disease on life), (3) *cause* (i.e., perceived causes of the disease), (4) *timeline* (i.e., perceived timeline of the disease; acute or chronic), (5) *cure/control* (i.e., perceived controllability over the disease; Leventhal et al., 1984, 2003), (6) *coherence* (i.e., perceived understanding of the disease; clear or confusing), (7) *emotional representations* (i.e., negative feelings caused by the disease such as anger and anxiety), and (8) *concerns* (i.e., levels of worry; Broadbent et al., 2006; Moss-Morris et al., 2002).

In light of similarities and discrepancies in disease characteristics of T2DM and MCI, there is a pressing need to understand and address illness perceptions about those conditions from the perspectives of affected individuals, as unique illness perceptions may be key factors for performing or maintaining self-management behaviors.

Although each dimension of illness perception plays a pivotal role in engaging in self-management behaviors, the chief among these may be the *control* and *coherence* dimensions in the context of disease characteristics (Gomersall et al., 2015; Wierenga, 2017). Persons with physical conditions may have a clearer understanding of their conditions or symptoms (Stallings, 2016) than those with cognitive conditions (Lin et al., 2012; Matchwick et al., 2014). A meta-analysis of chronic conditions (Hagger et al., 2017) found that perceived understanding and controllability over the conditions had direct or indirect effects on health outcomes such as role functioning (e.g., work adjustment, activities of daily living) and well-being (e.g., positive affect, general satisfaction). Although studies have investigated physical (Hagger et al., 2017) or cognitive conditions (Lin et al., 2012), they have not examined how illness perceptions are different or similar in the context of disease characteristics (physical or cognitive).

Therefore, exploring illness perceptions of T2DM and MCI, especially whether affected individuals believe their condition can be controlled or not (*control*) and whether the condition is understandable or confusing (*coherence*) to them, is paramount in developing effective interventions aiming at symptom or condition management in the context of disease characteristics.

With a particular focus on the dimensions of control and coherence, this study examined illness perceptions among persons with T2DM and MCI, treating each condition as an exemplar of late-life physical and cognitive health conditions. We hypothesized that, compared with persons with T2DM, those with MCI would be less likely to believe that their condition is controllable and understandable. We also investigated other dimensions of illness perception and possible moderation effects of age, education, and comorbid conditions in the relationship between disease characteristics (T2DM or MCI) and illness perceptions.

## Research Design and Methods

### Participants and Settings

This cross-sectional, descriptive study used existing baseline data collected from two independent studies. For the T2DM sample, we used the HABIT Study (hereafter referred to as HABIT), designed to improve medication adherence among persons with T2DM (NIH/NINR P01 NR010949). The Return of Amyloid Imaging Scan Results (RAISR) Study (hereafter referred to as RAISR) was used for the MCI sample, which explored the decision-making process of pursuing AD biomarker testing and reactions to the scan results among persons with MCI and their care partners (NIH/NIA R01 AG046906; Lingler et al., 2020).

In HABIT, persons with T2DM (*N* = 167) were recruited from outpatient clinical practice sites within the University of Pittsburgh Medical Center Health System. Individuals were eligible if they were (a) at least 40 years of age and in treatment for T2DM; (b) taking one or more medications prescribed by a physician; (c) self-managing their medications; (d) an English speaker, and (e) had access to a telephone. persons with T2DM were excluded if they were (a) had T2DM medications managed by others, (b) unwilling to use a medication diary or electronic event monitor, and (c) participated in other intervention studies.

The MCI sample (*N* = 90) was recruited from the University of Pittsburgh Alzheimer’s Disease Research Center (ADRC; NIH/NIA P50 AG005133). Individuals were eligible if they (a) were 50 years of age or older, (b) had an MCI diagnosis through a multidisciplinary consensus meeting at the ADRC based on the guidelines of MCI classification from the National Alzheimer’s Coordinating Center and the National Institute on Aging and Alzheimer’s Association (NIA-AA; Albert et al., 2011; Lopez et al., 1999; Snitz et al., 2018), (c) resided within 50 miles of the University of Pittsburgh, and (d) had a care partner (i.e., family member or kin-like friend). Excluded persons with MCI were (a) medically unstable or (b) had evidence of active, untreated primary psychiatric disorders (e.g., depression, anxiety disorder). As this investigation focused on late-life chronic conditions, persons with T2DM younger than 50 years of age (*n* = 20; 12%) were excluded from this analysis, so the analysis sample for persons with T2DM was 147. Care partner participants in RAISR were also excluded. This study was approved by the University of Pittsburgh.

### Measures

The Brief Illness Perception Questionnaire (Brief IPQ; Broadbent et al., 2006) comprises nine items asking about an individual’s perceptions of the health condition. Except for the *causality* item, all items, *consequences, timeline, personal control, treatment control, identity, concerns, coherence,* and *emotional representations*, were rated on an 11-point Likert scale from 0 (*not at all*) to 10 (*extremely*). We excluded the *causality* item because the item is an open-ended question. As suggested by Broadbent et al. (2006), the original term “illness” was replaced with “diabetes” and “my memory or thinking difficulties,” respectively. For example, *control* and *coherence* items were assessed using the following questions: “How much control do you feel you have over your ‘diabetes’ [HABIT] or ‘memory or thinking difficulties’ [RAISR]?” and “How well do you feel you understand your ‘diabetes’ [HABIT] or ‘memory or thinking difficulties’ [RAISR]?” The Brief IPQ has been widely used to investigate illness perceptions across chronic conditions (Al-Amer et al., 2016; Martinez et al., 2018; Perez, 2015; Saarti et al., 2016) and has demonstrated good test–retest reliability and validity (Broadbent et al., 2006, 2015).

Sociodemographic and clinical information included age, education, sex, race, marital status, and the number of comorbid conditions. In HABIT, baseline sociodemographic and clinical information was obtained using a standardized questionnaire developed through the Center for Research in Chronic Disorders (CRCD; Sereika & Engberg, 2006). In RAISR, sociodemographic and clinical information was abstracted from each consenting participant’s most recent ADRC record.

Because the two parent studies used different questionnaires for the number of comorbid conditions, several steps were taken to pool these data into one variable. First, the principal investigator (H. Kim) reviewed and identified similarities and differences in the items of the comorbid condition questionnaires. Next, each comorbid condition item in RAISR was linked to the CRCD Comorbidity Questionnaire (Sereika & Engberg, 2006) because the items in the CRCD questionnaire included more comorbid conditions. If there was a discrepancy between the forms, the items were dropped from the final form. For example, we dropped the “vitamin B12 deficiency” item because the ADRC form included this, whereas the CRCD form did not. Also, if the questionnaires investigated similar health conditions, we opted to use more generalized items to include all the related health conditions. For example, although the CRCD questionnaire has an item for overall heart conditions, the ADRC form had separate items for the heart conditions such as myocardial infarction, congestive heart failure, atrial fibrillation, and angina. If a person with MCI answered they had been diagnosed with any of these heart conditions, we coded this participant as diagnosed with “heart conditions” using the CRCD questionnaire. After re-coding the comorbid condition data based on the CRCD questionnaire, a doctorally prepared statistician reviewed this procedure to verify the reliability of the data.

### Statistical Analysis

All analyses were performed using SPSS Statistics Version 28.0 (IBM Corporation, Armonk, NY). The level of statistical significance for two-sided hypothesis testing was set at less than .05. Prior to analysis, each of the data sets from HABIT (*N* = 147) and RAISR (*N* = 90) were screened using descriptive and exploratory methods separately and collectively for anomalies (e.g., outliers, missing data, violations of statistical assumptions). The baseline data sets from both studies were pooled for analysis based on sociodemographic and clinical factors, and each dimension of illness perception.

Through the data screening, we excluded one individual (0.68%) from HABIT because this person did not complete the Brief IPQ. Except for the *timeline* and *coherence* dimensions, statistical assumptions were met for each dimension of illness perception in the model. Because the dimensions of *timeline* and *coherence* were severely negatively skewed, these variables were reflected, and then a square root transformation of the scales was applied. We analyzed both original and transformed variables and obtained similar results; therefore, the original variables (untransformed) were used in our analysis.

First, group comparisons were performed for all sociodemographic characteristics (i.e., age, sex, race, education, and marital status) by participants’ disease group (T2DM [0] or MCI [1]) using independent *t* tests (for continuous-type variables) or chi-square tests of independence (for categorical variables). If significant differences were found, the variables were controlled for as covariates in the main analyses. We also explored each dimension of illness perception using descriptive statistics (i.e., mean, standard deviation [*SD*], and median) between persons with T2DM and those with MCI. Second, we performed a multivariate analysis of covariance (MANCOVA) to compare the set of illness perceptions simultaneously based on the eight dimensions of the Brief IPQ between persons with T2DM and those with MCI. Accordingly, hierarchical multiple linear regression analysis was conducted to examine the similarities and differences in each dimension of illness perception among persons with T2DM and those with MCI. For each dimension of illness perception, three successive linear regression models were estimated. In block one, a model with only covariates (i.e., age, sex, race, marital status, and the number of comorbid conditions) was estimated. In block two, a primary independent variable (disease group; T2DM or MCI) was added. In the final block, interaction terms between the disease group variable and targeted covariates (i.e., age, education, and the number of comorbid conditions) were added to explore the possible interactions between the disease group and targeted covariates on each dimension of illness perception. To minimize possible multicollinearity, variables of age, education, and the number of comorbid conditions were centered when creating the interaction terms.

## Results

### Sample Characteristics

The final pooled sample (*N* = 236) included 146 persons with T2DM (61.9%) and 90 (38.1%) persons with MCI (Table 1). Persons with T2DM and those with MCI differed significantly across sociodemographic characteristics, except for the number of comorbid conditions (*p* = .089). MCI participants were older and had more years of education, with an average of 72.5 (*SD* = 8.77) years of age and 16.5 (*SD* = 2.55) years of education, than T2DM participants who were an average of 64.4 (*SD* = 8.65) years of age and had 14 (*SD* = 2.82) years of education. Although six-tenths of persons with MCI (*n* = 84, 57.5%) were women, less than half (*n* = 62, 42.5%) were women in the T2DM sample. Most participants were White in both T2DM (*n* = 98, 67.1%) and MCI (*n* = 83, 92.2%) samples. Over half of the T2DM sample (*n* = 75, 51.4%) and three-quarters of MCI sample (*n* = 69, 76.7%) were married or living as married.

**Table 1.** Sociodemographic and Clinical Characteristics of T2DM and MCI Participants (*N* = 236)

Variable	Total (*N* = 236)	T2DM sample (*n* = 146)	MCI sample (*n* = 90)	Test statistic	*p*
Age (years), mean ± *SD*	67.48 ± 9.54	64.38 ± 8.65	72.51 ± 8.77	*t*(234) = −6.98	<.001
Education (years), mean ± *SD*	14.92 ± 2.98	13.97 ± 2.82	16.48 ± 2.55	*t*(234) = −6.89	<.001
Sex: male, *n* (%)	116 (49.2)	62 (42.5)	54 (60.0)	*x* ^2^ (1) = 6.849	.009
Race: White, *n* (%)	181 (76.7)	98 (67.1)	83 (92.2)	*x* ^2^ (1) = 19.624	<.001
Marital status: married/living with a partner, *n* (%)	144 (61.0)	75 (51.4)	69 (76.7)	*x* ^2^ (1) = 14.979	<.001
Number of comorbid conditions,^a^ mean ± *SD* (range min.–max.)	4.98 ± 1.69 (1–11)	4.84 ± 1.58 (1–11)	5.22 ± 1.85 (2–10)	*t*(234) = −1.71	.089

*Notes*: max. = maximum; MCI = mild cognitive impairment; min. = minimum; *SD* = standard deviation; T2DM = type 2 diabetes.

^a^T2DM and MCI were calculated.

### Illness Perceptions of Late-Life Physical and Cognitive Health Conditions: T2DM Versus MCI

Supplementary Table 1 reports the descriptive statistics for each illness perception among persons with T2DM and those with MCI. The highest mean score in both groups was on the timeline (mean ± *SD* = 8.77 ± 2.01 [T2DM]; mean ± *SD* = 8.29 ± 2.41 [MCI]), indicating that all participants perceived their conditions as chronic rather than acute. The identity dimension was the lowest mean score in the T2DM group (mean ± *SD* = 4.10 ± 2.49), suggesting persons with T2DM may experience fewer disease-related symptoms. In the MCI group, the consequences dimension had the lowest mean score (mean ± *SD* = 4.24 ± 2.20), indicating that persons with MCI may perceive the diagnosis or symptoms of MCI had little impact on their life. The MANCOVA test was statistically significant (*F* [80, 1800] = 2.50, *p* < .001, Pillai’s trace = 0.380; results not shown), indicating that persons with T2DM and MCI have significantly different illness perceptions about their conditions.

Results from hierarchical linear regression models assessing the differences between persons with T2DM and MCI for each dimension of illness perception considering covariates are summarized in Tables 2–5 and Supplementary Figure 1.

**Table 2.** Linear Hierarchical Regression Model of Disease Group (T2DM or MCI) as a Predictor of Consequences and Timeline (*N* = 236)

Block	Predictor	Consequences						Timeline					
		Model 1		Model 2		Model 3		Model 1		Model 2		Model 3	
		Unstandardized regression coefficients											
		*b*	*p*	*b*	*p*	*b*	*p*	*b*	*p*	*b*	*p*	*b*	*p*
1	(Constant)	7.460	<.001	7.461	<.001	6.710	.001	8.547	<.001	6.794	<.001	5.464	.003
	Age (years)	−0.027	.129	−0.027	.161	−0.028	.259	−0.009	.566	0.008	.635	0.030	.158
	Male^a^	0.151	.664	0.151	.665	0.175	.614	−0.288	.349	−0.282	.352	−0.278	.358
	White^b^	−0.366	.400	−0.366	.402	−0.428	.329	1.017	.008	1.068	.005	1.041	.007
	Education (years)	−0.105	.071	−0.105	.089	−0.114	.135	0.027	.600	0.075	.163	0.062	.355
	Married/living with a partner^c^	<0.001	.999	−0.001	.999	−0.028	.942	−0.173	.595	0.012	.970	0.024	.942
	Comorbid condition^d^	0.137	.163	0.137	.166	0.340	.012	−0.058	.506	−0.037	.668	−0.019	.874
2	MCI ^e^			0.001	.999	0.020	.962			−0.945	.008	−0.903	.013
3^f^	MCI × age					−0.008	.831					−0.062	.065
	MCI × education					0.062	.629					0.085	.450
	MCI × comorbid condition					−0.432	.027					−0.049	.771
	*R* ^2^	0.045		0.045		0.068		0.036		0.065		0.082	
	Adjusted *R*^2^	0.020		0.015		0.026		0.011		0.036		0.041	

*Notes*: MCI = mild cognitive impairment; T2DM = type 2 diabetes.

^a^Women was treated as the reference category for sex.

^b^Non-White was treated as the reference category for race.

^c^The combined category of never married, widowed, separated, or divorced was treated as reference category for marital status.

^d^T2DM and MCI were calculated.

^e^T2DM was treated as the reference category for disease group.

^f^For interactions, age, education, and number of comorbid conditions were mean centered due to multicollinearity.

#### Consequences

Persons with T2DM and with MCI did not have a significantly different perception of the consequences of their health conditions (Table 2). However, the interaction of participants’ disease group (T2DM or MCI) and the number of comorbid conditions was significant (*b* = −0.432, *p* = .027) for the perceived consequences of health conditions (Supplementary Figure 1A). This indicates that MCI participants felt that their memory difficulties have more impact on their lives when they have less comorbid health conditions, whereas an opposite relationship was found among T2DM participants (*b* = 0.340, *p* = .012).

#### Timeline

A significant association between the disease group and timeline perception (*b* = −0.945, *p* = .008; Table 2) suggests that participants with MCI perceived their condition as being less chronic compared with those with T2DM. This association remained significant when the interactions were added to the model (*b* = −0.903, *p* = .013; Table 2).

#### Personal control and treatment control

As seen in Table 3, participants’ disease groups were significantly associated with both personal control (*b* = −0.943, *p* = .009) and treatment control (*b* = −1.619, *p* < .001), and remained significant when the interaction terms were added to the model (personal control: *b* = −0.893, *p* = .016; treatment control: *b* = −1.435, *p* < .001). These findings suggest that participants with MCI felt that their cognitive changes are not likely to be controlled by either their effort or medical treatment compared with their T2DM counterparts. We found a significant interaction between the disease group and education on treatment control (*b* = −0.270, *p* = .011; Supplementary Figure 1B). This suggests that among persons with MCI, participants with more years of education were likely to believe that their cognitive changes could not be controlled through medical treatment (e.g., medication taking) compared to those with fewer years of education. Yet, among persons with T2DM, there was no relationship between years of education and the treatment control variable (*b* = 0.107, *p* = .087).

**Table 3.** Linear Hierarchical Regression Model of Disease Group (T2DM or MCI) as a Predictor of Personal Control and Treatment Control (*N* = 236)

Block	Predictor	Personal control						Treatment control					
		Model 1		Model 2		Model 3		Model 1		Model 2		Model 3	
		Unstandardized regression coefficients											
		*b*	*p*	*b*	*p*	*b*	*p*	*b*	*p*	*b*	*p*	*b*	*p*
1	(Constant)	7.801	<.001	6.052	<.001	5.107	.006	11.633	<.001	8.630	<.001	6.275	<.001
	Age (years)	0.002	.881	0.019	.254	0.036	.098	−0.035	.024	−0.006	.718	0.012	.555
	Male^a^	−0.117	.706	−0.111	.716	−0.125	.683	−0.540	.072	−0.530	.064	−0.578	.042
	White^b^	−0.205	.596	−0.153	.688	−0.165	.669	0.075	.842	0.163	.648	0.065	.855
	Education (years)	−0.045	.386	0.003	.953	0.015	.822	−0.074	.136	0.008	.878	0.107	.087
	Married/living with a partner^c^	−0.452	.170	−0.267	.422	−0.243	.466	−0.304	.340	0.014	.964	0.059	.849
	Comorbid condition^d^	−0.114	.194	−0.093	.284	−0.156	.193	−0.096	.256	−0.060	.455	−0.079	.472
2	MCI^e^			−0.943	.009	−0.893	.016			−1.619	<.001	−1.435	<.001
3^f^	MCI × age					−0.040	.248					−0.027	.389
	MCI × education					−0.016	.887					−0.270	.011
	MCI × comorbid condition					0.129	.454					0.049	.757
	*R* ^2^	0.032		0.061		0.068		0.277		0.404		0.438	
	Adjusted *R*^2^	0.007		0.032		0.027		0.053		0.137		0.156	

*Notes*: MCI = mild cognitive impairment; T2DM = type 2 diabetes.

^a^Women was treated as the reference category for sex.

^b^Non-White was treated as the reference category for race.

^c^The combined category of never married, widowed, separated, or divorced was treated as reference category for marital status.

^d^T2DM and MCI were calculated.

^e^T2DM was treated as the reference category for disease group.

^f^For interactions, age, education, and number of comorbid conditions were mean centered due to multicollinearity.

#### Identity

Participants’ disease group was the most significant predictor of the identity perception (*b* = 0.987, *p* = .012; Model 2, *b* = 0.922, *p* = .020; Model 3; Table 4), indicating that persons with MCI felt that they experienced more disease-related symptoms (e.g., forgetfulness) than their T2DM counterparts.

**Table 4.** Linear Hierarchical Regression Model of disease Group (T2DM or MCI) as a Predictor of Identity and Concerns (*N* = 236)

Block	Predictor	Identity						Concerns					
		Model 1		Model 2		Model 3		Model 1		Model 2		Model 3	
		*b*											
		*b*	*p*	*b*	*p*	*b*	*p*	*b*	*p*	*b*	*p*	*b*	*p*
1	(Constant)	5.146	<.001	6.977	<.001	7.233	<.001	3.260	.063	9.332	<.001	9.774	<.001
	Age (years)	−0.004	.817	−0.022	.237	−0.023	.319	0.002	.916	−0.056	.011	−0.081	.004
	Male^a^	−0.101	.763	−0.107	.746	−0.067	.838	−0.016	.972	−0.036	.929	−0.002	.996
	White^b^	−0.306	.465	−0.360	.385	−0.359	.387	−0.132	.809	−0.312	.533	−0.365	.466
	Education (years)	−0.046	.406	−0.096	.100	−0.155	.033	0.044	.544	−0.122	.085	−0.128	.142
	Married/living with a partner^c^	−0.020	.956	−0.213	.553	−0.248	.488	0.686	.142	0.044	.920	−0.009	.983
	Comorbid condition^d^	0.073	.438	0.052	.582	0.191	.137	0.202	.105	0.130	.253	0.399	.010
2	MCI^e^			0.987	.012	0.922	.020			3.273	<.001	3.246	<.001
3^f^	MCI × age					−0.012	.735					0.053	.228
	MCI × education					0.199	.104					0.020	.892
	MCI × comorbid condition					−0.305	.100					−0.563	.012
	*R* ^2^	0.013		0.041		0.066		0.024		0.196		0.223	
	Adjusted *R*^2^	−0.013		0.011		0.025		−0.002		0.172		0.188	

*Notes:* MCI = mild cognitive impairment; T2DM = type 2 diabetes.

^a^Women was treated as the reference category for sex.

^b^Non-White was treated as the reference category for race.

^c^The combined category of never married, widowed, separated, or divorced was treated as reference category for marital status.

^d^T2DM and MCI were calculated.

^e^T2DM was treated as the reference category for disease group.

^f^For interactions, age, education, and number of comorbid conditions were mean centered due to multicollinearity.

#### Concerns

As indicated in Table 4, we also found a significant relationship between the disease group and perceived concerns about the conditions (*b* = 3.273, *p* < .001; Model 2, *b* = 3.246, *p* < .001; Model 3). This suggests that persons with MCI were more worried about their disease-related symptoms than those with T2DM. We found an interaction between the disease group and the number of comorbid conditions on concerns (*b* = −0.563, *p* = .012; Supplementary Figure 1C), indicating having fewer comorbid conditions was associated with more concerns about cognitive changes among persons with MCI. Conversely, among persons with T2DM, those with more comorbid conditions had more concerns about their diabetes condition (*b* = 0.399, *p* = .010).

#### Coherence

As reported in Table 5, participants’ perceived understanding of their conditions or disease-related symptoms was significantly associated with the disease group (*b* = −1.265, *p* = .001), and this association remained significant when interactions were added to the model (*b* = −1.312, *p* = .001). The findings demonstrate that MCI participants believed they had a lower understanding of their disease than T2DM participants. Older age was significantly associated with a better perceived understanding of the disease in MCI participants (*b* = 0.080, *p* = .026; Supplementary Figure 1D), although no association was found between age and perceived understanding of the condition in the T2DM participants.

**Table 5.** Linear Hierarchical Regression Model of Disease Group (T2DM or MCI) as a Predictor of Coherence and Emotional Representations (*N* = 236)

Block	Predictor	Coherence						Emotional representations					
		Model 1		Model 2		Model 3		Model 1		Model 2		Model 3	
		Unstandardized regression coefficients											
		*b*	*p*	*b*	*p*	*b*	*p*	*b*	*p*	*b*	*p*	*b*	*p*
1	(Constant)	9.336	<.001	6.989	<.001	8.630	<.001	13.156	<.001	9.101	<.001	7.722	<.001
	Age (years)	−0.026	.123	−0.003	.855	−0.031	.165	−0.075	<.001	−0.036	.050	−0.012	.614
	Male^a^	−0.485	.140	−0.478	.138	−0.485	.130	−0.666	.062	−0.652	.052	−0.673	.045
	White^b^	−0.510	.215	−0.441	.273	−0.408	.312	0.192	.665	0.312	.455	0.294	.486
	Education (years)	0.021	.700	0.085	.134	0.106	.130	−0.051	.391	0.060	.308	0.079	.285
	Married/living with a partner^c^	−0.423	.226	−0.174	.617	−0.187	.590	−0.615	.104	−0.186	.609	−0.151	.677
	Comorbid condition ^d^	0.134	.150	0.162	.077	0.134	.281	−0.084	.401	−0.036	.702	−0.125	.337
2	MCI ^e^			−1.265	.001	−1.312	.001			−2.186	<.001	−2.113	<.001
3^f^	MCI × age					0.080	.026					−0.057	.130
	MCI × education					−0.121	.307					−0.027	.830
	MCI × comorbid condition					0.073	.684					.182	.330
	*R* ^2^	0.059		0.103		0.128		0.124		0.229		0.240	
	Adjusted *R*^2^	0.034		0.076		0.089		0.101		0.206		0.206	

*Notes*: MCI = mild cognitive impairment; T2DM = type 2 diabetes.

^a^Women was treated as the reference category for sex.

^b^Non-White was treated as the reference category for race.

^c^The combined category of never married, widowed, separated, or divorced was treated as reference category for marital status.

^d^T2DM and MCI were calculated.

^e^T2DM was treated as the reference category for disease group.

^f^For interactions, age, education, and number of comorbid conditions were mean centered due to multicollinearity.

#### Emotional representations

The disease group variable was a robust predictor of participants’ disease-related emotional representations (e.g., anger, anxiety; *b* = −2.186, *p* < .001; Table 5), indicating that persons with MCI felt fewer disease-related negative feelings than persons with T2DM. The dimension of emotional representations remained significant (*b* = −2.113, *p* < .001; Table 5) when interactions were added to the regression model, but no interactions were found.

## Discussion and Implications

The present study examined illness perceptions among persons with T2DM and those with MCI by treating each condition as an exemplar of age-related physical and cognitive health conditions. Given that research on chronic conditions moves toward identifying the factors related to secondary prevention strategies, this study of T2DM and MCI propels our understanding of disease-specific illness perceptions, potential facilitators, or barriers to self-management, in the context of disease characteristics, physical or cognitive. Undoubtedly, both T2DM and MCI participants believed their conditions would be long-lasting rather than short-term regardless of the disease characteristics. Our analyses supported the primary hypotheses that persons with MCI perceive the condition as less susceptible to control (either through personal strategies or medical treatment) and less likely to make sense to them compared with those with T2DM. This study also revealed partial support for the exploratory aim, the moderating role of age, education, and comorbid conditions in the relationship between participants’ disease group (T2DM or MCI) and illness perceptions.

The findings of lower levels of perceived control over the conditions among persons with MCI than those with T2DM are congruent with previous studies in the context of disease characteristics. For example, persons with hypertension (Stallings, 2016) and those with heart failure (Wierenga, 2017) were likely to believe their conditions could be controlled through their own strategies or medical treatment. Conversely, persons with MCI and AD (Lin & Heidrich, 2012; Matchwick et al., 2014) had varied control perceptions due to the uncertainty of the conditions. Although the ways to diagnose (e.g., fasting plasma glucose test, hemoglobin A1C test) and manage (e.g., lifestyle modification, taking medications) T2DM are robust, there is currently no diagnostic test, and no condition-specific medications or interventions to restore cognitive function for MCI, making it reasonable that persons with MCI would have a lower sense of control over their condition. This may be the primary reason that our MCI participants had significantly lower control perceptions over their condition than their T2DM counterparts. Given that the perceived controllability has been identified as a driver of problem-focused coping strategies (Hagger et al., 2017), our findings suggest that persons with T2DM may tend to adopt more problem-solving coping behaviors such as regular medical checkups and asking for support from health care providers as compared to those with MCI. However, there is a strong consensus among researchers and clinicians about the significance of a regular cognitive assessment along with regular physical activity for cognitive health in MCI (Petersen et al., 2018), indicating our findings should be further examined using an in-depth approach to identify the factors that affected control perceptions among persons with T2DM and with MCI.

Interestingly, in the MCI participants, more years of education were associated with a lower treatment control perception, yet this association was not significant in the T2DM participants. Information about MCI continues to evolve rapidly, especially biomarkers of ADRD for early detection and treatment. Persons with MCI who attained higher education may actively seek up-to-date information through various resources so that they may come to believe that any treatments might not be effective for their cognitive function. A meta-analysis study found that low control perceptions negatively affected psychological distress and disease state across chronic health conditions (Hagger et al., 2017), suggesting further research is needed to investigate how education is related to perceived treatment control among persons with MCI. Of note, caution is required in interpreting this finding as our participants with MCI reported relatively high levels of educational attainment, with an average of 16 years of education.

Consistent with prior investigations of physical and cognitive conditions (Lin et al., 2012; Stallings, 2016; Wierenga, 2017), we observed that persons with MCI had a lower perceived understanding of their condition than those with T2DM, which was another hypothesis of this study. Also, such illness coherence perception in our MCI sample supports previous qualitative research that the diagnosis or cognitive symptoms do not make sense to persons with MCI (Gomersall et al., 2015, 2017). Given that prior research (Gomersall et al., 2015, 2017; Lin et al., 2012; Stallings, 2016; Wierenga, 2017) investigated either physical or cognitive condition, comparing perceived understanding of the conditions among persons with T2DM and with MCI strengthens this study. This pattern of perceived understanding about the diagnosis or memory symptoms among persons with MCI could potentially reflect their unmet information needs regarding the management of the symptoms and the prospect of progression to ADRD.

Unlike MCI, T2DM is a relatively specific condition in the context of its diagnostic process and management of blood glucose, which may be associated with T2DM participants’ better perceived understanding of their condition or its symptoms than their MCI counterparts. Literature suggests that the extent of an individual’s perceived understanding of the disease has been associated with approaches to managing the disease or symptoms (Hagger & Orbell, 2022; Hagger et al., 2017). Yet, it should be noted that a higher perceived understanding of the condition may not necessarily lead to desirable self-management behaviors in the context of MCI. For example, although persons with T2DM may adhere to the treatment regimen if the condition makes sense, those with MCI who feel their cognitive changes are confusing may have more doctor’s visits to check their cognitive status or actively seek the most up-to-date information. Future research is needed on how illness coherence perception affects subsequent self-management behaviors in the context of the disease characteristics.

We also found that individuals who were older were more likely to believe they had a better understanding of the condition than those younger among MCI participants but not among T2DM participants. Older individuals may be more exposed to cognitive impairment than younger in that cognitive changes increase with aging. However, in a nationwide study of 6,141 older adults with and without cognitive impairment (Lee et al., 2016), younger participants had higher knowledge of ADRD than older participants, regardless of their cognitive impairment. Moreover, among persons with MCI, age was not a significant factor in predicting knowledge of ADRD in this population-based study, suggesting that our older MCI participants’ better perceived understanding of cognitive changes may not necessarily mean that they have better knowledge of their condition than younger MCI participants. Research is needed to investigate how the perceived understanding of the condition is associated with objective knowledge among persons with MCI.

It is noteworthy that other dimensions of illness perceptions were also significantly different between persons with T2DM and those with MCI in the form of main effects or interaction effects, which was not our primary hypothesis. In particular, the interaction effects between the number of comorbid conditions and certain illness perceptions, consequences, and concerns, should be further examined. In our analysis, among persons with MCI, less comorbid conditions were associated with more worries and a higher perceived impact of MCI on life, which was the opposite finding among their T2DM counterparts. One possible explanation is that persons with MCI with less comorbid conditions may pay more attention to their cognitive changes than those with more comorbid conditions. The opposite perception among persons with T2DM may be attributable to high comorbidity risks of T2DM if not managed well. Given that older adults with multiple conditions had complex illness perceptions intertwined with other factors (Schüz et al., 2012), illness perceptions should be investigated in the context of multimorbidity.

Our study has some limitations. First, our sample of participants with T2DM and MCI was drawn from two existing datasets, which may lead to study bias due to the mixing of different datasets. To minimize this issue, we controlled for sociodemographics (i.e., age, sex, race, years of education, and marital status) and the number of comorbid conditions which were identified as covariates. Despite the fact that the time since diagnosis and current success in disease management may be essential factors of illness perception, we could not include these variables because the parent studies did not collect the information. In addition, although some participants might have been diagnosed with both T2DM and MCI conditions, we were not able to assess how having both conditions affected the perceptions of T2DM or MCI we targeted due to the nature of the secondary analysis. Second, the use of self-reported questionnaires can be an issue. For example, responses may be influenced by social desirability; that is, participants might answer the items considered more socially accepted. Lastly, because participants had an average of five comorbid conditions, they might have multiple illness perceptions or perceptions about conditions other than T2DM or MCI, which might affect the study results (Schüz et al., 2012) even though the general term “illness” in the Brief IPQ was replaced with condition-specific terms, “diabetes” or “my memory or thinking difficulties” in each parent study.

Nevertheless, the findings of this study provided clinical and research implications in that, to our knowledge, this is the first theory-driven study that identified similarities and differences in illness perceptions in chronic disorders in the context of disease characteristics (physical vs cognitive). As T2DM and MCI are common late-life chronic conditions, our sample represents key targets for treatments to delay or stop disease progression. Assessment of unique illness perceptions depending on the disease characteristics may be an essential underpinning of patient counseling for symptom and disease management in clinical and research settings. Illness perceptions in other late-life conditions should also be explored to ensure that our findings can replicate.

## Supplementary Material

igad027_suppl_Supplementary_Material
