# Supplementary material for: Do Individuals Aged 50 or Older View Cognitive Conditions Differently Than Physical Conditions? Evidence From a Pooled Analysis of Illness Perceptions in Type 2 Diabetes and Mild Cognitive Impairment
Source: Innov Aging. 2023 Apr 13;7(3):igad027. doi: 10.1093/geroni/igad027 (PMC10148452; doi:10.1093/geroni/igad027)
Supplement: igad027_suppl_Supplementary_Material [file igad027_suppl_supplementary_material.docx]

| **Supplementary Table S1.** Illness perceptions in T2DM and MCI participants (N = 236) | | | |
| --- | --- | --- | --- |
| Dimension of illness perception  (possible score: 0–10) | Total  (N = 236) | T2DM sample  (n = 146) | MCI sample  (n = 90) |
|  | Mean (±SD)/median rating | | |
| Consequences | 4.55 ± 2.49/4 | 4.74 ± 2.64/5 | 4.24 ± 2.20/4 |
| Timeline | 8.59 ± 2.18/10 | 8.77 ± 2.01/10 | 8.29 ± 2.41/10 |
| Personal control | 6.24 ± 2.20/6 | 6.60 ± 2.08/7 | 5.66 ± 2.26/6 |
| Treatment control | 7.32 ± 2.17/8 | 7.97 ± 1.96/8 | 6.26 ± 2.09/7 |
| Identity | 4.26 ± 2.35/4 | 4.10 ± 2.49/4 | 4.52 ± 2.10/4 |
| Concerns | 5.39 ± 3.10/6 | 4.45 ± 2.99/5 | 6.93 ± 2.64/8 |
| Coherence | 7.69 ± 2.36/8 | 8.16 ± 2.36/9 | 6.91 ± 2.17/7 |
| Emotional representations | 6.36 ± 2.65/7 | 7.28 ± 2.34/8 | 4.86 ± 2.43/5 |
| *Note.* SD = standard deviation; MCI = mild cognitive impairment; T2DM = type 2 diabetes; Min. = minimum; Max. = maximum | | | |

| 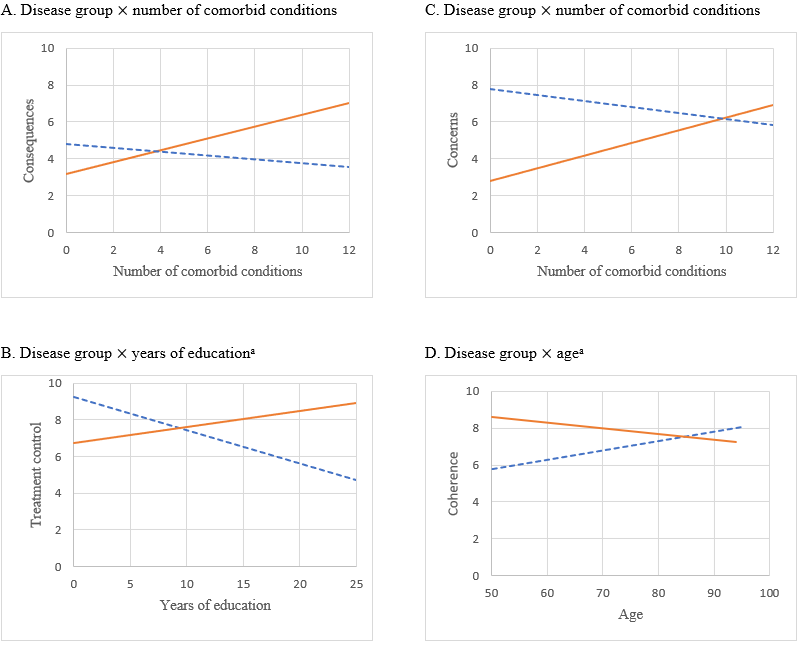  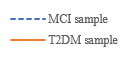 |
| --- |

**Supplementary Figure S1.** Interaction effects between disease group (T2DM or MCI) and covariates (i.e., age, years of education, and the number of comorbid conditions) on illness perceptions. T2DM = type 2 diabetes; MCI = mild cognitive impairment

^a^No interaction effects were observed among persons with T2DM.
